# Clinical Relevance of Plasma Endogenous Tissue-Plasminogen Activator and Aortic Valve Sclerosis: Performance as a Diagnostic Biomarker

**DOI:** 10.3389/fcvm.2020.584998

**Published:** 2020-10-14

**Authors:** Zhongli Chen, Ying Shen, Qiqi Xue, Bo Wen Lin, Xiao Yan He, Yi Bo Zhang, Ying Yang, Wei Feng Shen, Ye Hong Liu, Ke Yang

**Affiliations:** ^1^Department of Cardiology, Ruijin Hospital, Shanghai Jiao Tong University School of Medicine, Shanghai, China; ^2^Department of Cardiology, Shanghai East Hospital, Tongji University School of Medicine, Shanghai, China; ^3^Department of Endocrinology, The Second People's Hospital of Yunnan Province, Kunming, China

**Keywords:** calcific aortic valve disease, aortic valve sclerosis, tissue plasminogen activator, diagnostic value, biomarker

## Abstract

**Background:** Aortic valve sclerosis (AVSc), a common precursor to calcific aortic valve disease, may progress into advanced aortic stenosis with hemodynamic instability. However, plasma biomarkers of such a subclinical condition remain lacking. Since impaired fibrinolysis featuring dysregulated tissue plasminogen activator (t-PA) is involved in several cardiovascular diseases, we investigated whether endogenous t-PA was also associated with AVSc.

**Methods:** Plasma t-PA levels were measured in 295 consecutive patients undergoing standard echocardiography and Doppler flow imaging. Multiple logistic regression analyses were used to assess the association between t-PA and AVSc. Receiver operating characteristic curve analysis was performed for determining the diagnostic value of t-PA for AVSc. The performance of adding t-PA to clinical signatures of AVSc was evaluated. Concentration of t-PA was assessed in human sclerotic and non-sclerotic aortic valves by histology and immunohistochemistry analysis.

**Results:** Plasma t-PA was higher in patients with AVSc than in non-AVSc counterparts (median, 2063.10 vs. 1403.17 pg/mL, *p* < 0.01). C-statistics of plasma t-PA for discriminating AVSc was 0.698 (95%CI: 0.639–0.758). The performance of t-PA for identifying AVSc was better among male and non-hypertensive patients [C-statistics (95%CI): 0.712 (0.634–0.790) and 0.805 (0.693–0.916), respectively]. Combination of t-PA and clinical factors improved classification of the patients (category-free NRI: 0.452, *p* < 0.001; IDI: 0.020, *p* = 0.012). The concentration of t-PA was three times higher in sclerotic compared to non-sclerotic aortic valves.

**Conclusion:** Elevated circulating t-PA level confers an increased risk for AVSc. Further prospective studies with larger sample size are needed to examine if t-PA could serve as a diagnostic clinical marker for AVSc.

## Introduction

Calcific aortic valve disease (CAVD) represents the most prevalent valve disease worldwide (1), but it is usually detected at an advanced stage (known as aortic stenosis), with severe clinical symptoms and hemodynamic instability. Accumulating evidence revealed that aortic valve sclerosis (AVSc), a non-uniform thickening of valve leaflets without impairment of leaflet excursion or a significant transvalvular pressure gradient (2), is rather common in asymptomatic populations (3), with a prevalence ranging from 30 to 40% in general population and increased occurrence in the elderly (4, 5). Early identification of AVSc at its subclinical stage could improve risk stratification and enable timely intervention and disease control in clinical practice. However, scarce biomarkers were available regarding the identification of AVSc.

Although previous studies unveiled that CAVD shares certain pathophysiological similarities with atherosclerosis in terms of inflammation and dyslipidemia (6, 7), randomized trials did not prove a convincing protective effect of lowering low-density lipoprotein cholesterol (LDL-C) therapy on retarding aortic stenosis (8–10). These observations suggest that different mechanisms exist during these two disease processes, and the strategy of targeting risk factors of atherosclerosis may not be likely to reverse advanced aortic stenosis. Thus, investigations of novel pathogenesis and biomarkers in the initial stage of CAVD before the occurrence of clinical severe conditions are urgently required.

Over the past decades, increasing researches have demonstrated an extensive cross-talk between inflammation and coagulation in stenotic valve lesions, which is attributed, at least partly, to calcification and mineralization of the aortic valve leaflets (11, 12). Therefore, coagulation or fibrinolysis regulators may be potential participants in the development and progression of AVSc. Tissue plasminogen activator (t-PA) has been proved as a risk factor in various cardiovascular diseases (13–16). Secreted by endothelia cells, t-PA facilitates the conversion of plasminogen to plasmin (17) and plays a pivotal role in the homeostasis of blood coagulation/fibrinolysis and matrix regulation (18, 19). Although the acute release of t-PA contributes to thrombus dissolution, disrupted fibrinolysis featuring chronic elevation of plasma t-PA might reflect presence of long-lasting endothelial injury, which is a remarkable early pathological alteration in AVSc (13, 20). Additionally, t-PA has been shown to act as a cytokine to activate profound receptor-mediated signaling events (21) such as inflammatory response to tissue ischemic injury in various models (22–24). These findings highlight that t-PA might also be implicated in AVSc. In this study, we aimed to explore the association of plasma t-PA levels with AVSc and evaluate the performance of t-PA in identification of AVSc.

## Methods

The protocol was approved by the Shanghai Jiao Tong University ethic committee and conducted in accordance with the Declaration of Helsinki. All patients gave written informed consent.

### Study Population

A total of 571 patients who underwent coronary angiography/intervention because of typical angina symptom and/or electrocardiographic ST-T wave changes from September 2017 to July 2018 were screened from the database of Shanghai Rui Jin Hospital PCI Outcome Program. Among them, 445 patients underwent standard two-dimensional echocardiography and Doppler flow imaging according to the recommendations of the American Society of Echocardiography (25). All measurements were based on the observations of two independent experienced investigators. Data on demographics, clinical, laboratorial and angiographic features, and in-hospital management were collected.

**Figure 1:**
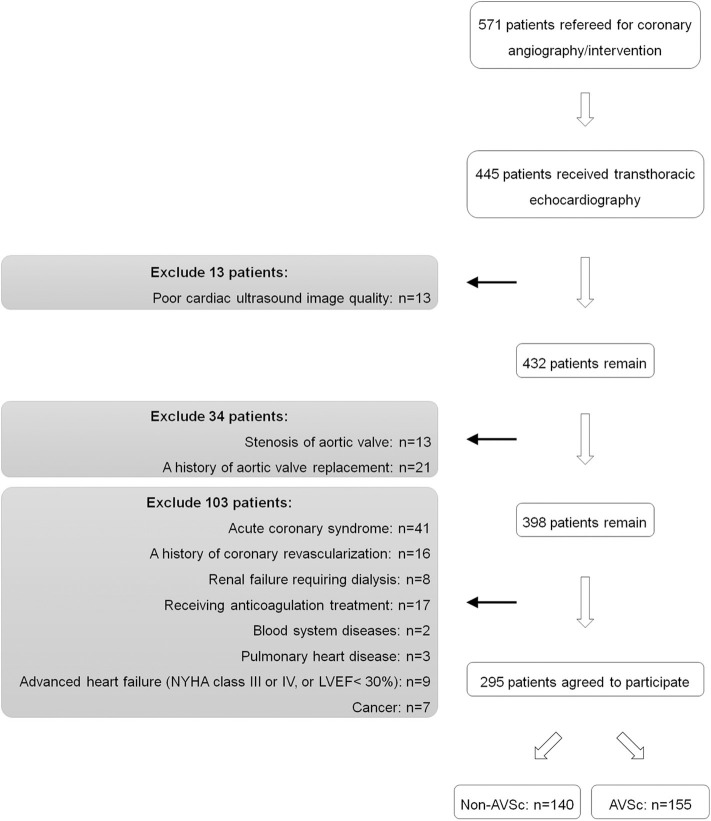
Flowchart of patient enrollment.

432 patients who had high-quality images of transthoracic echocardiography and Doppler flow data were recruited. We excluded patients with history of aortic valve replacement (*n* = 21) or aortic stenosis (*n* = 13) as these conditions did not comply with the proper diagnosis of AVSc. We also excluded patients with acute coronary syndrome (*n* = 41), history of coronary artery bypass grafting (*n* = 16), renal failure requiring dialysis (*n* = 8), anticoagulation treatment (*n* = 17), blood system diseases (*n* = 2), pulmonary heart disease (*n* = 3), advanced heart failure (NYHA functional class III or IV, or left ventricular ejection fraction <30%) (*n* = 9) and cancer (*n* = 7), because these factors are likely to interfere t-PA measurement. Thus, the remaining 295 eligible patients were included in the cross-sectional study analysis, including 155 patients with and 140 patients without AVSc (Figure 1).

### Definitions

**Figure 2:**
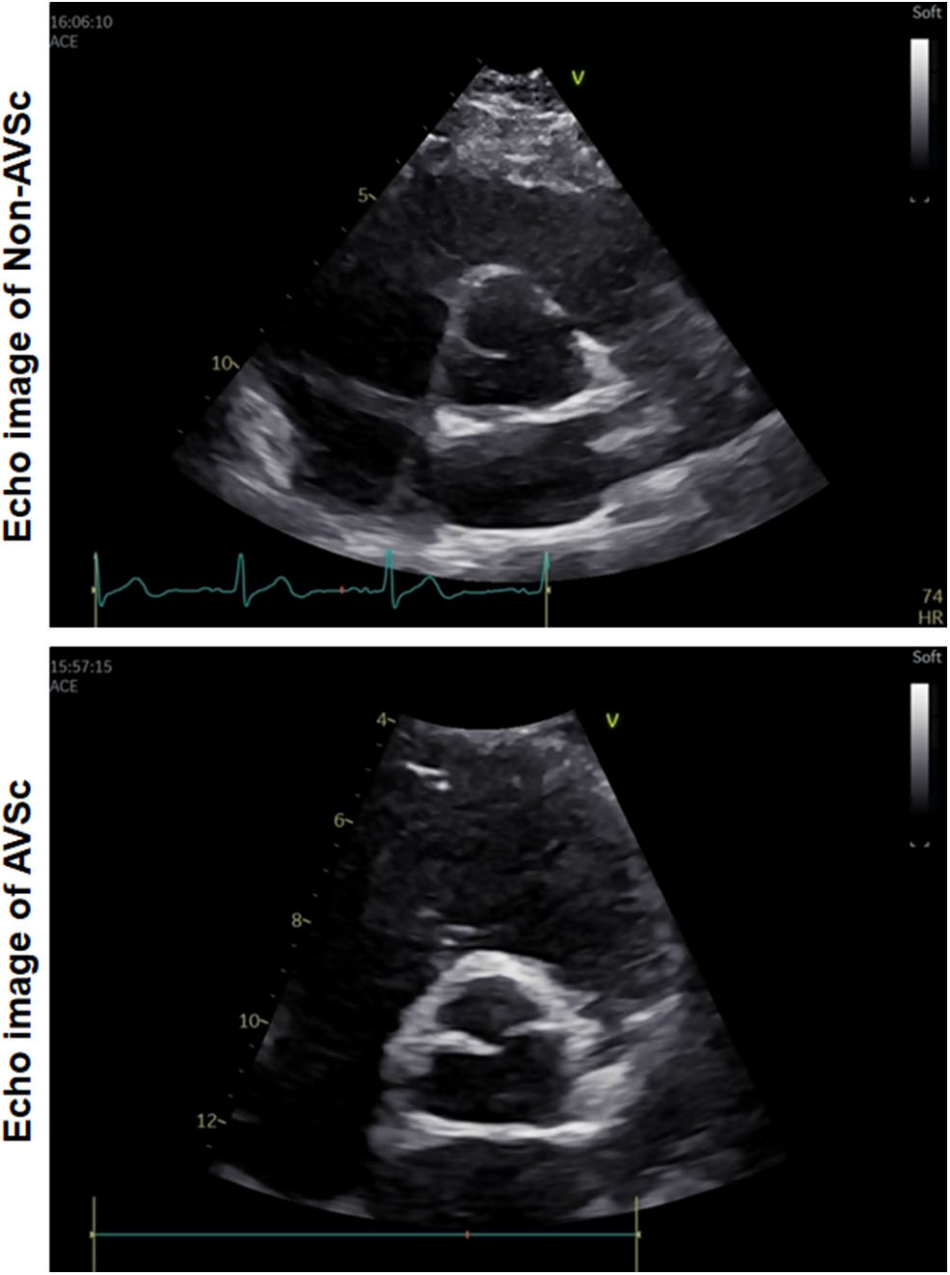
Representative echo images of aortic valve. Upper: non-AVSc; Bottom: AVSc.

AVSc was defined as non-transparent aortic valve cusps with focal areas of mild thickening or increased echogenicity without motive restriction and a peak flow velocity across the aortic valve <2.0 m/s (Figure 2) (26). Hypertension was diagnosed in accordance with ESC/ESH guidelines (27). Coronary artery disease was diagnosed if ≥50% luminal diameter stenosis in at least one major epicardial coronary artery was detected, according to the lesion classification scheme of the American College of Cardiology/American Heart Association (28). Renal function was assessed based on estimated glomerular filtration rate (eGFR), which was calculated using the Modification of Diet in Renal Disease equation.

### Determination of Plasma t-PA

Fasting venous blood samples were taken from all participants within 24 h of echocardiographic examination. All blood samples were transferred at the freezing temperature and centrifuged within 30 min at 3,000 rpm for 15 min to obtain cell-free plasma. All samples were stored at −80°C before analysis. Plasma level of t-PA was measured using an ELISA kit (Tissue Plasminogen Activator (TPA) Human SimpleStep ELISA Kit; Abcam Cat No: ab190812) according to the product instructions.

### Human Tissue Samples and Immunohistochemistry

Human sclerotic aortic valve tissues were obtained from three patients with AVSc who underwent aortic valve replacement within 3 years because of aortic dissection (*n* = 2) or moderate aortic regurgitation with deteriorating renal function (*n* = 1). The non-sclerotic aortic valve tissues were obtained from three explanted hearts of cardiac transplant recipients.

The concentration of t-PA in human sclerotic and non-sclerotic aortic valves was assessed by histological and immunochemical analysis. Briefly, after an overnight-fixation in 4% paraformaldehyde, samples were sliced into serial cryosections (5 μm thickness). Sections were stained using hematoxylin and eosin (H&E), Alizarin Red S and Masson trichrome, respectively. For t-PA detection, sections were incubated with anti-t-PA antibody (1:50) (Rabbit monoclonal, Abcam: #ab157469) at 4°C for 12 h and then incubated with horseradish peroxidase (HRP)-conjugated secondary antibodies (1:100) (CST: #3900S) at room temperature for 2 h. Image of all sections were photographed through a microscope (Zeiss Microsystems).

### Statistical Analysis

Continuous variables are expressed as mean with standard deviation (SD) if data were normally distributed, then presented as median with interquartile ranges (IQRs). Categorical data are summarized as proportions and frequencies. Continuous variables between two groups were compared using *t*-test (Student's *t*-test or Welch's *t*-test) or non-parametric test while categorical variables in two groups were compared by Chi-squared tests. Spearman rank correlation was conducted to evaluate the correlations between clinical parameters and t-PA concentrations. Due to its right skew, t-PA concentration was expressed as log 10 transformed per SD in logistic regression model analysis and divided into ordinal variable based on its tertile using the lowest tertile as reference for comparison. Univariate logistic regression models were performed to examine possible factors associated with AVSc. Multivariable logistic regression analysis was conducted to assess the independent association between t-PA and AVSc. In Model 1, demographic features were included as covariates. To detect whether the association between t-PA levels and AVSc could be affected by other confounders, we further adjusted age, gender, smoking, diabetes, coronary artery disease, hypertension, lipid profiles, C-reactive protein (CRP), gamma-glutamyltransferase (γGT), eGFR, and levels of phosphorus in Model 2. Odds ratios with 95% confidence intervals (CI) were reported. Receiver operating characteristic (ROC) curves were constructed to estimate the performance of t-PA concentration for discriminating AVSc in the whole study population and different subgroups. The optimal cut-off value of t-PA concentration was calculated based on the highest Youden index (sensitivity + specificity – 1). C-statistics with 95% CIs were reported. To distinguish the representative variables for AVSc, the least absolute shrinkage and selection operator (LASSO) approach was utilized for feature selection (29–31). The selected variables were confirmed based on lambda with one standard error from the minimum using 5-fold internal cross-validation. T-PA and other four clinical features including age, coronary artery disease, high density lipoprotein-cholesterol (HDL-C), eGFR were selected. A clinical model using the four clinical variables was constructed by multivariate logistic regression. C-statistics and brier score were respectively calculated for evaluating discrimination and calibration of the clinical model both before and after the addition of t-PA. Category-free net reclassification improvement (NRI) and integrated discrimination improvement (IDI) indexes were calculated to assess the added value of t-PA in reclassification of the patients.

Data were analyzed with the use of SPSS 22.0., statistical packages R glmnet (version 3.0-2) (The R Foundation; http://www.r-project.org; version 3.6.1), and two-sided *p*-value <0.05 was considered statistically significant.

## Results

### Baseline Clinical Characteristics

Compared with non-AVSc patients, those with AVSc were older and had a higher proportion of hypertension and coronary artery disease (all *p* < 0.01). In addition, plasma levels of HDL-C, phosphorus and eGFR were lower but CRP and γGT were higher in patients with AVSc (all *p* < 0.05). However, the two groups did not differ with respect to gender distribution, percentage of diabetes, smokers, plasma level of triglyceride, total cholesterol, LDL-C, and fast blood glucose. No significant difference was observed in terms of left ventricular geometry and function between the two groups. Medications including statin and antiplatelet treatments were also similar (Table 1).

**Table 1:** Baseline characteristics of non-AVSc and AVSc patients.

	**Non-AVSc (*n* = 140)**	**AVSc (*n* = 155)**	***P*-value**
Age (years)	60 (56–66)	75 (69–80)	<0.001
Male, *n* (%)	72 (51.4%)	94 (62.6%)	0.111
Smoking, *n* (%)	35 (25.0%)	33 (21.3%)	0.450
Coronary artery disease, *n* (%)	90 (64.3%)	130 (83.9%)	<0.001
Diabetes, *n* (%)	43 (31.7%)	48 (31.0%)	0.962
Hypertension, *n* (%)	92 (65.7%)	120 (77.4%)	0.026
Systolic BP (mmHg)	132 ± 18	140 ± 21	<0.001
Diastolic BP (mmHg)	77 ± 11	75 ± 11	0.09
Fast blood glucose (mmol/L)	5.00 (4.50–5.65)	5.01 (4.56–5.88)	0.712
Triglyceride (mmol/L)	1.45 (1.20–2.06)	1.32 (1.02–2.09)	0.230
Total cholesterol (mmol/L)	3.93 (3.37–4.56)	3.87 (3.14–4.58)	0.218
HDL-C (mmol/L)	1.13 (0.99–1.29)	1.04 (0.91–1.20)	0.005
LDL-C (mmol/L)	2.31 (1.72–2.93)	2.17 (1.73–2.90)	0.581
C-reactive protein (mg/L)	1.01 (0.51–2.41)	1.52 (0.68–3.56)	0.01
eGFR (ml/min/1.73 m^2^)	87.13 ± 17.83	71.64 ± 17.24	<0.001
γ-GT (IU/L)	18.00 (12.00–30.25)	22.00 (16.00–29.00)	0.025
Calcium(mg/dL)	2.21 ± 0.20	2.19 ± 0.11	0.294
Phosphorus(mg/dL)	1.17 ± 0.18	1.11 ± 0.20	0.004
Pulmonary systolic pressure (mmHg)	21.50 ± 2.42	22.10 ± 1.98	0.079
Mean aortic valvegradient (mmHg)	3.76 ± 1.23	3.81 ± 1.16	0.122
Peak aortic transvalvularvelocity (m/s)	1.22 ± 0.24	1.26 ± 0.23	0.579
Left ventricular			
mass index (g/m^2^)	96.9 ± 25.4	96.7 ± 24.8	0.694
ESVI (ml/m^2^)	20.6 ± 14.5	21.4 ± 15.8	0.312
EDVI (ml/m^2^)	51.8 ± 12.8	52 ± 14.7	0.325
Ejection fraction (%)	55 ± 7	54 ± 7	0.258
Medications			
statin, *n* (%)	84 (60.0%)	101 (65.2%)	0.360
anti-platelet, *n* (%)	76 (54.3 %)	92 (59.4%)	0.380

*Normally distributed variates: mean ± SD, %; skewed variates: median (interquartile range). AVSc, aortic valve sclerosis; BP, blood pressure; HDL-C, high-density lipoprotein cholesterol; LDL-C, low-density lipoprotein cholesterol; eGFR, estimated glomerular filtration rate; γ-GT, gamma-glutamyl transferase; ESVI, end-systolic volume index; EDVI: end-diastolic volume index*.

### Association of Plasma t-PA Levels With AVSc

**Figure 3:**
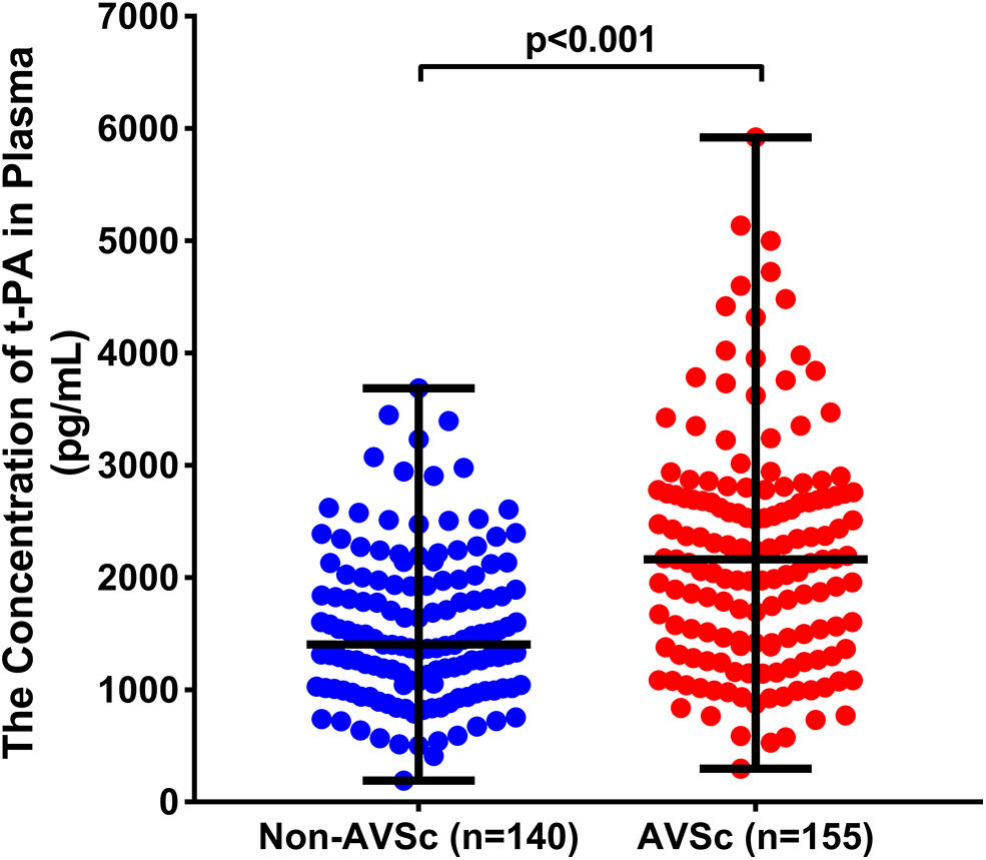
Comparison of plasma t-PA between patients with and without AVSc. t-PA levels (pg/mL) were significantly higher in AVSc (median = 2063.10 pg/mL [IQR 1403.54.89–2731.46 pg/mL; range 297.18–5922.58 pg/mL], red dots) than in non-AVSc patients (median = 1403.17 pg/mL [IQR percentile 1026.50–1976.95 pg/mL; range 191.11–3687.57 pg/mL], blue dots) (*p* < 0.001).

Plasma levels of t-PA were significantly higher in patients with AVSc [median = 2063.10 pg/mL (IQR 1403.54.89–2731.46 pg/mL)] than in non-AVSc counterparts [median = 1403.17 pg/mL (IQR percentile 1026.50–1976.95 pg/mL, *p* < 0.001)] (Figure 3).The Spearman rank correlation analysis showed that plasma t-PA level correlated positively with age, status of coronary artery disease, as well as levels of CRP, γGT and triglyceride while it was inversely related to HDL-C levels and eGFR (Supplementary Table 1). In univariate analysis, plasma t-PA level as both continuous variable [OR 95%CI: 2.091 (1.592–2.746), *p* < 0.001] and categorical variable was associated with AVSc [tertile 3rd to tertile 1st OR 95%CI: 5.328 (2.885–9.840), *p* < 0.001]. Such an association remained significant even after adjusting for either demographic features alone [OR 95%CI: 1.753 (1.251–2.458), *p* = 0.001] or demographic features, clinical and laboratory cofounders [OR 95%CI: 1.582 (1.117–2.242), *p* = 0.010]. An independent association between the highest tertile of t-PA level and AVSc was also observed after full adjustment [OR 95%CI: 2.533 (1.122–5.716), *p* = 0025] (Table 2).

**Table 2:** Association between plasma t-PA levels and AVSc.

	**Crude model OR (95% CI)**	***P*-value**	**Model 1 OR (95% CI)**	***P*-value**	**Model 2 OR (95% CI)**	***P*-value**
Lg (t-PA) per SD	2.091 (1.592–2.746)	<0.001	1.753 (1.251–2.458)	0.001	1.582 (1.117–2.242)	0.010
t-PA 1st tertile	1(ref)		1 (ref)		1 (ref)	
t-PA 2nd tertile	1.592 (0.898–2.824)	0.111	1.631 (0.775–3.432)	0.197	1.458 (0.665–3.196)	0.346
t-PA 3rd tertile	5.328 (2.885–9.840)	<0.001	3.238 (1.490–7.036)	0.003	2.533 (1.122–5.716)	0.025

*t-PA concentrations are analyzed as a log transformed continuous variable and a categorical variable using the lowest tertile as reference. Crude model: Unadjusted model. Model 1: adjusted for age and gender. Model 2: adjusted for age, gender, smoking, diabetes, coronary artery disease, hypertension, lipid profiles, CRP, γGT, eGFR, levels of phosphorus. When t-PA as a continuous variable, OR is shown as log 10 per 1 SD (standard deviation); OR, odds ratio*.

**Figure 4:**
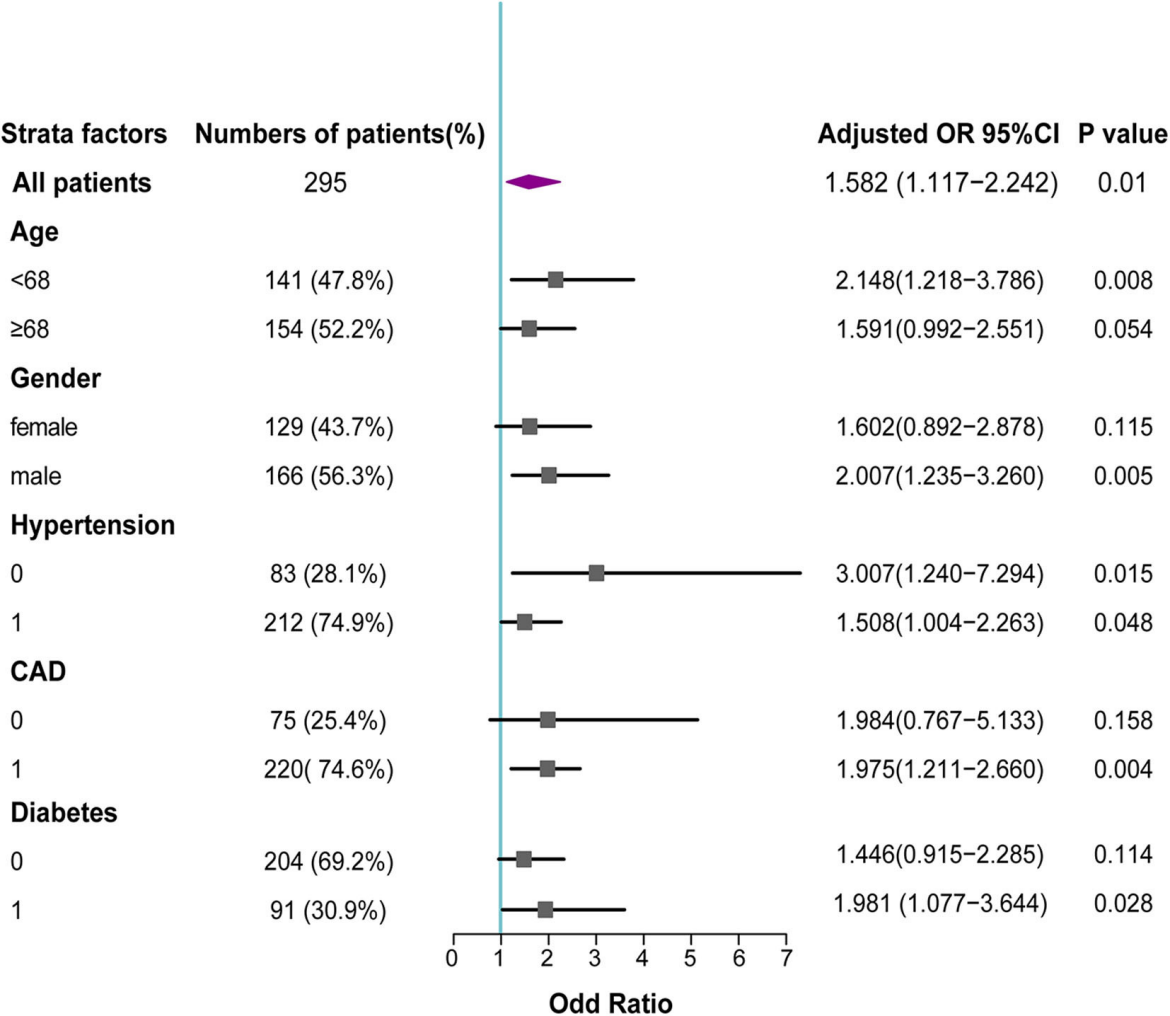
Forest plot for association between t-PA levels and AVSc in different subgroups. In subgroup analysis, the adjusted odd ratios with 95% CI revealed that elevated t-PA levels remained associated with AVSc in various subgroups, and add more risk for AVSc in male patients, those with younger age, diabetes or without hypertension.

Furthermore, higher plasma levels of t-PA still tended to be associated with higher risks of AVSc in different subgroups after adjusting for other confounding factors. In particular, t-PA levels appeared to add more risk for AVSc in male patients, those with younger age, diabetes, or those without hypertension (Figure 4).

### Performance of Circulating t-PA for Discrimination of AVSc

**Figure 5:**
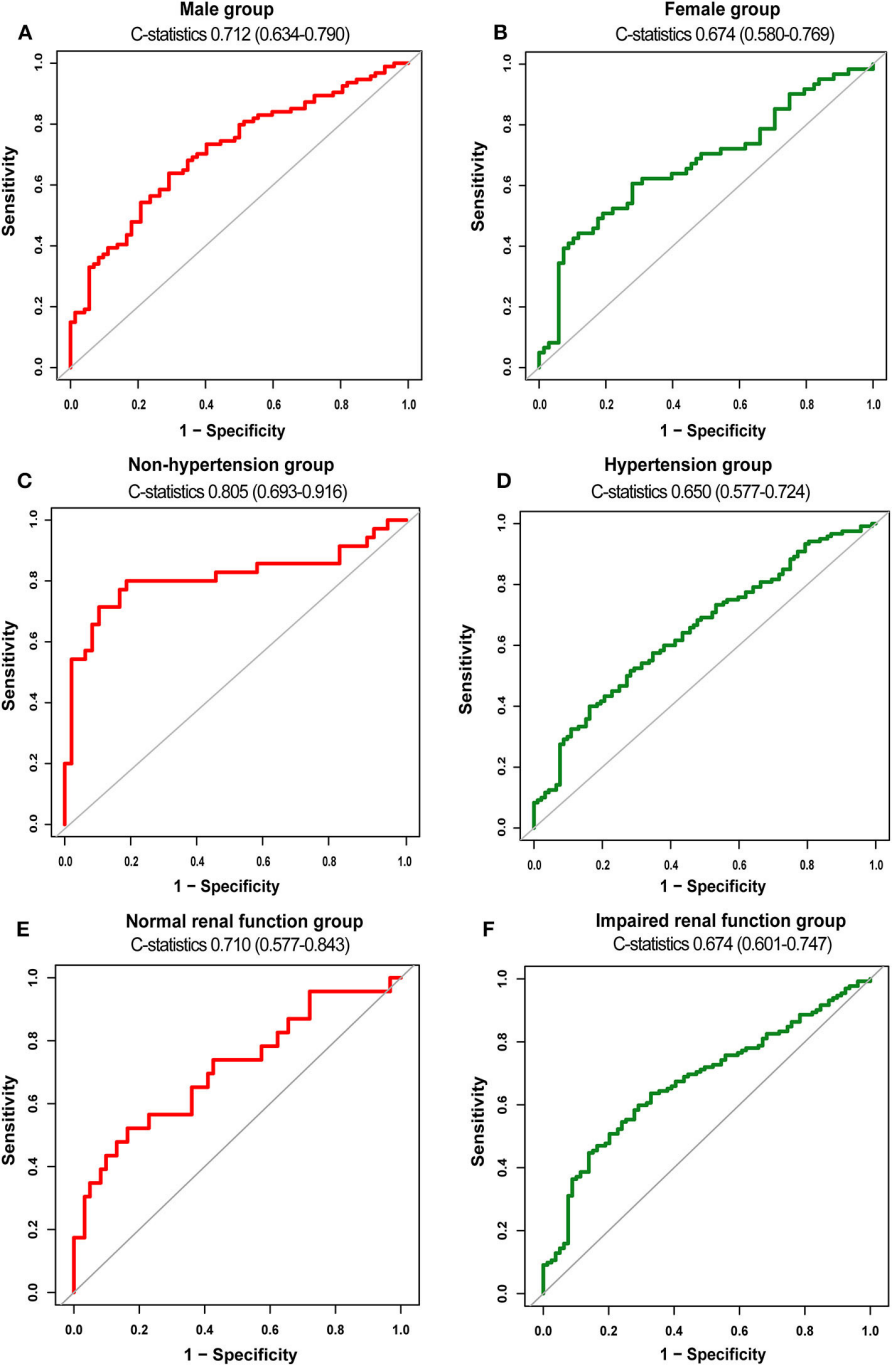
Diagnostic value of t-PA for AVSc in different subgroups. ROC curves of t-PA as a biomarker for AVSc among male patients **(A)** and female group **(B)** non-hypertensive group **(C)** and hypertensive group **(D)**, normal renal function group **(E)**, and impaired renal function group **(F)**.

To evaluate the discriminability of plasma t-PA levels for AVSc, we performed ROC analysis and calculated the C-statistics. In our whole study population, t-PA held a C-statistics of 0.698 (0.639–0.758), with a cut-off value of 1845.79 pg/ml (specificity 0.707; sensitivity 0.626). The performance of t-PA in discriminating AVSc in different subgroups is summarized in Table 3. Notably, t-PA showed a stronger discriminability for AVSc among non-hypertensive [C-statistics, 0.805 (95% CI, 0.693–0.916)] and male patients [C-statistics, 0.712 (95% CI 0.634–0.790)] as well as those with normal renal function [C-statistics, 0.710 (95% CI 0.577–0.843)] (Figure 5).

**Table 3:** Performance of t-PA in discriminating AVSc among different subgroups.

	**C-statistic (AUC-ROC)**	**Sensitivity**	**Specificity**	**Positive predictive value**	**Negative predictive value**	**Cut off (pg/mL)**
Overall	0.698(0.639–0.758)	0.626	0.707	0.703	0.631	1845.796
Younger age	0.663(0.547–0.779)	0.433	0.856	0.448	0.848	2194.702
Older age	0.640(0.527–0.754)	0.776	0.483	0.866	0.333	1391.271
Female	0.674(0.580–0.769)	0.607	0.721	0.661	0.671	1845.796
Male	0.712(0.634–0.790)	0.638	0.708	0.741	0.600	1936.596
Non-hypertension	0.805(0.693–0.916)	0.800	0.813	0.757	0.848	1863.247
Hypertension	0.650(0.577–0.724)	0.400	0.837	0.762	0.517	2283.940
Non-diabetes	0.700(0.628–0.772)	0.664	0.680	0.696	0.647	1842.298
Diabetes	0.693(0.584–0.802)	0.479	0.861	0.793	0.597	2152.725
Non-CAD	0.675(0.637–0.814)	0.600	0.780	0.577	0.796	1946.231
CAD	0.699(0.629–0.768)	0.631	0.689	0.746	0.564	1845.796
Normal renal function	0.710 (0.577–0.843)	0.522	0.836	0.546	0.823	2199.840
Impaired renal function	0.674 (0.601–0.747)	0.447	0.861	0.843	0.482	2253.148

*AUC, area under the curve; ROC, receiver operating characteristic; Younger age, <68 years old; Older age, ≥68 years old; Normal renal function, eGFR <90 mL/min/1.73 m; Impaired renal function, eGFR ≥90 mL/min/1.73 m; CAD, coronary artery disease*.

For detecting the potential role of t-PA in assisting clinical factors for AVSc classification, a LASSO logistic regression was initially performed for selecting the representative signatures of AVSc among all clinical and laboratory features. Remarkably, based on lambda with one standard error from the minimum, t-PA (lg/SD) was selected as one of the representative variables for AVSc, along with another four clinical variables, including age, presence of coronary artery disease, HDL-C as well as eGFR (Supplementary Figure 1). These factors were also well-established clinical variables associated with AVSc. Afterwards, a basic clinical model incorporating the four clinical factors was established by simple logistic regression. The addition of t-PA to the clinical model appeared to offer better discrimination and calibration than the basic clinical model alone, with a slightly higher C-statistics of 0.895 and a lower brier score of 0.129. More importantly, inclusion of plasma t-PA levels allowed a significant reclassification of the patients, with IDI of 0.020 (95% CI: 0.004–0.035) and category-free NRI of 0.452 (95% CI: 0.229–0.674) (Table 4).

**Table 4:** Performance of t-PA in improving diagnosis of AVSc.

	**C-statistics (95% CI)**	**Brier**	**Category-free NRI (95%CI)**	***P*-value**	**IDI (95%CI)**	***P*-value**
Clinical model	0.890(0.853–0.927)	0.134	reference model		reference model	
Clinical model + t-PA (lg/SD)	0.895(0.859–0.931)	0.129	0.452(0.229–0.674)	p <0.001	0.020(0.004–0.035)	0.012

*Variables in Clinical model: coronary artery disease, age, high-density lipoprotein-cholesterol, estimated glomerular filtration rate. NRI, net reclassification improvement; IDI, integrated discrimination improvement*.

### Increased Concentration of t-PA in Human Sclerotic Aortic Valve Tissues

**Figure 6:**
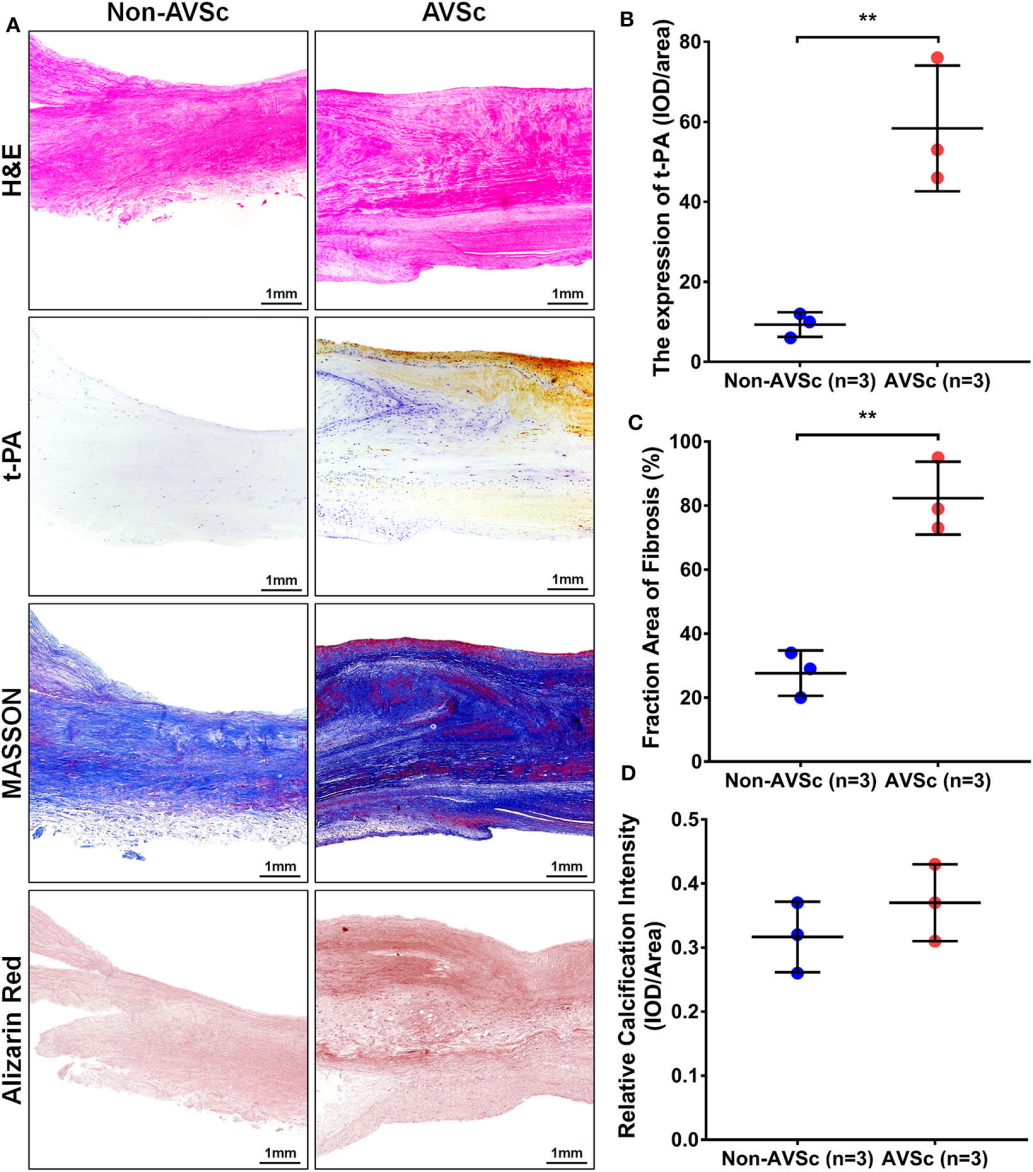
Levels of t-PA in sclerotic and non-sclerotic aortic valves. **(A)** Hematoxylin-eosin (HE) staining showed that aortic valve leaflets were thicker in AVSc than in non-AVSc group. Masson and alizarin red staining also revealed relatively stronger fibrosis and degree of calcification in sclerotic aortic valve leaflets. The immuno-histochemical staining revealed higher levels of t-PA in sclerotic aortic valves compared with non-sclerotic aortic valves. **(B)** The levels of t-PA were three times higher in sclerotic than in non-sclerotic aortic valves and the fibrosis degree of valve were two times stronger in the sclerotic aortic valves. The calcification degree of sclerotic valve was slightly stronger than the non-sclerotic valves, although without statistical significance. **(C)** Quantification of the extent of fibrosis based on Masson staining. **(D)** Quantification of calcific intensity by integrated optical density (IOD), and the calcific intensity was expressed as IOD/area. **p < 0.01.

Hematoxylin-eosin (HE), Masson, and alizarin red staining revealed that aortic valve leaflets were thicker and more fibrotic or calcified in AVSc patients compared to non-AVSc counterparts (Figures 6A,C,D). Immunohistochemical staining showed that t-PA level was three times higher in the AVSc group than in the non-AVSc group (Figures 6A,B).

## Discussion

The results of this study showed that elevated t-PA levels in plasma were associated with higher risk of AVSc, and may hold potential in AVSc discrimination especially for male, non-hypertensive and normal renal function patients. The concentration of t-PA was increased in sclerotic aortic valves.

Growing publications indicated that impaired fibrinolysis was associated with CAVD (11, 32). However, to date, most studies regarding this topic mainly focused on aortic stenosis. Whether the alteration of blood coagulation and fibrinolysis was the product of adverse valve condition or reflected the early stage of CAVD has not been reported. In this study, the association between higher plasma t-PA levels and AVSc persisted even after adjusting for multiple variables including demographic features, clinical history and other laboratory factors. Interestingly, the risk of AVSc increased by 58.2% for every unit increase in plasma t-PA (lg per SD), even after full adjustment. Our finding is different from prior work by Kochtcebane et al. which implied that t-PA levels in stenotic or calcified human aortic valves was relatively normal (33). The reason for this remains unclear but may be more likely explained by different study populations and distinct reference and sample source.

Moreover, beyond the relatively satisfactory discriminability in the whole study population, plasma t-PA tended to be an indicator of AVSc among patients without hypertension. Over the past years, accumulating evidence revealed that hypertension was more prevalent in patients with CAVD (34, 35), and was considered as a risk factor for aortic valve disease (36, 37). Nevertheless, AVSc and even aortic stenosis also occurred in individuals without hypertension (38, 39). And for these patients without traditional risk factors, elevated plasma t-PA levels held higher discriminating power for early identification of AVSc in clinical scenarios. However, the clinical and pathological relevance of elevated t-PA levels and progression of AVSc in this population still needs to be investigated in a long-term follow-up study. In addition, our study showed a relatively higher diagnostic value of plasma t-PA for AVSc among male patients. Early identification of male patients with AVSc may be clinically meaningful as they tend to develop moderate and severe aortic stenosis and have a higher risk of all-cause mortality (40). Similarly, although the association of valve calcification with renal dysfunction is well-recognized (41–43), our results suggest that t-PA appears to display modest discriminability for AVSc, with reduced plasma t-PA being of potential to exclude risks of AVSc among patients with normal renal function.

In this study, we applied the machine learning approach LASSO regression for feature selection, which shrinks some variables coefficients to zero to ruling out redundant or irrelevant features or features that are strongly correlated and generate easily interpretable models with concise information (29). Interestingly, besides the established risk factors such as age, coronary artery disease, HDL-C and eGFR, t-PA was also selected as a representative feature for AVSc. Although the method did not reflect biological importance, it still indicates that t-PA might be an effective factor at least for AVSc classification. In line with that, when adding t-PA to the basic clinical model, more patients were better reclassified. This suggests that determination of plasma t-PA levels could provide additional information beyond clinical risk factors in terms of reclassification of patients into more appropriate diagnostic groups.

The mechanism of the development of AVSc is likely complex and multi-factorial. t-PA has been considered as a cytokine triggering profound intracellular signaling events and modulating inflammation response by interacting with membrane receptors such as low-density lipoprotein receptor—related protein-1 (LRP-1) (21). Animal experiments have identified t-PA as a regulator of inflammatory response. Several models have shown that t-PA knock-out caused less hepatic fibrosis with decreased T cells and relevant cytokines (44), less neutrophil influx into the interstitial area and quicker recovery of renal function than wildtype mice (23). Meanwhile, t-PA induced microglial activation with generation of an inflammatory response in the ischemic brain and adherence of polymorphonuclear neutrophils to the endothelium under flow conditions (22, 24). These findings support a notion that t-PA may induce inflammation via cytokine release, which in turn facilitate endothelium injury and contribute to the development of AVSc. Another possible mechanism can be attributed to the influence of t-PA on extracellular matrix remodeling. Specifically, t-PA increased the biological activity of metalloproteinase (MMP) 2 and 9 both *in vivo* and *in vitro* (18, 19). In accordance, given the essential role of MMPs activation in promoting valvular remodeling and inflammation in CAVD (45), it is reasonable to hypothesize that t-PA may contribute to AVSc through activation of MMPs.

Aortic stenosis is the most common form of valvular heart disease in the elderly population and occurs frequently in conjunction with coronary artery disease (46). In this study, elevated t-PA levels were associated with presence of coronary artery disease and old age, and both conditions tended to be more prevalent among patients with AVSc. Nevertheless, after adjusting for these confounding factors, higher t-PA levels remained independent risk factor for AVSc either as a continuous variable or within the highest tertile. This suggests that t-PA might be of interest regarding the pathophysiology of AVSc.

## Limitations

Admittedly, several limitations exist in our study. First, as a single-center study, patient recruitment, staff characteristics, and departmental protocols might add a limitation to the universality of our results. Secondly, our diagnosis of AVSc is based on transthoracic echocardiography, and AVSc has not been assessed quantitatively by cardiac computed tomography. Though cardiac CT has not been recommended by guidelines as a routine test, it has been shown to display the burden of calcium weight in the aortic valve area. Additionally, despite careful adjustment for the major known confounders, unspecified elements could also interfere with our findings. Finally, as observational cross-sectional study, the prognostic value and causal relationship cannot be concluded. Further basic researches and clinical investigation are still required.

## Conclusions

The current study indicates that elevated plasma t-PA confers an increased risk for AVSc. This observation could extend our understanding of the pathophysiology of CAVD. Further prospective studies with larger sample size are needed to examine if t-PA is able to serve as a diagnostic clinical marker for AVSc.

## Data Availability Statement

The raw data supporting the conclusions of this article will be made available by the authors, without undue reservation.

## Ethics Statement

The studies involving human participants were reviewed and approved by Ruijin Hospital Ethics Committee, Shanghai Jiao Tong University School of Medicine. The patients/participants provided their written informed consent to participate in this study.

## Author Contributions

All authors contributed to conception and design, acquisition of data or analysis and interpretation of data, drafting the article or revising it critically for important intellectual content, and gave final approval of the version to be published. Individual significant contributions include: YL and KY provided study design, interpretation, and edited manuscript, were the guarantors of this work and took full responsibility for the work. ZC, YS, and QX performed data collection and wrote the manuscript. XH, YZ, YY, and ZC analyzed the data. WS contributed to interpretation, drafting, and editing the manuscript.

## Conflict of Interest

The authors declare that the research was conducted in the absence of any commercial or financial relationships that could be construed as a potential conflict of interest.
