# Supplementary material for: Clinical Relevance of Plasma Endogenous Tissue-Plasminogen Activator and Aortic Valve Sclerosis: Performance as a Diagnostic Biomarker
Source: Front Cardiovasc Med. 2020 Oct 14;7:584998. doi: 10.3389/fcvm.2020.584998 (PMC7591748; doi:10.3389/fcvm.2020.584998)
Supplement: Supplementary file 1 [file Data_Sheet_1.docx]

Supplementary Material


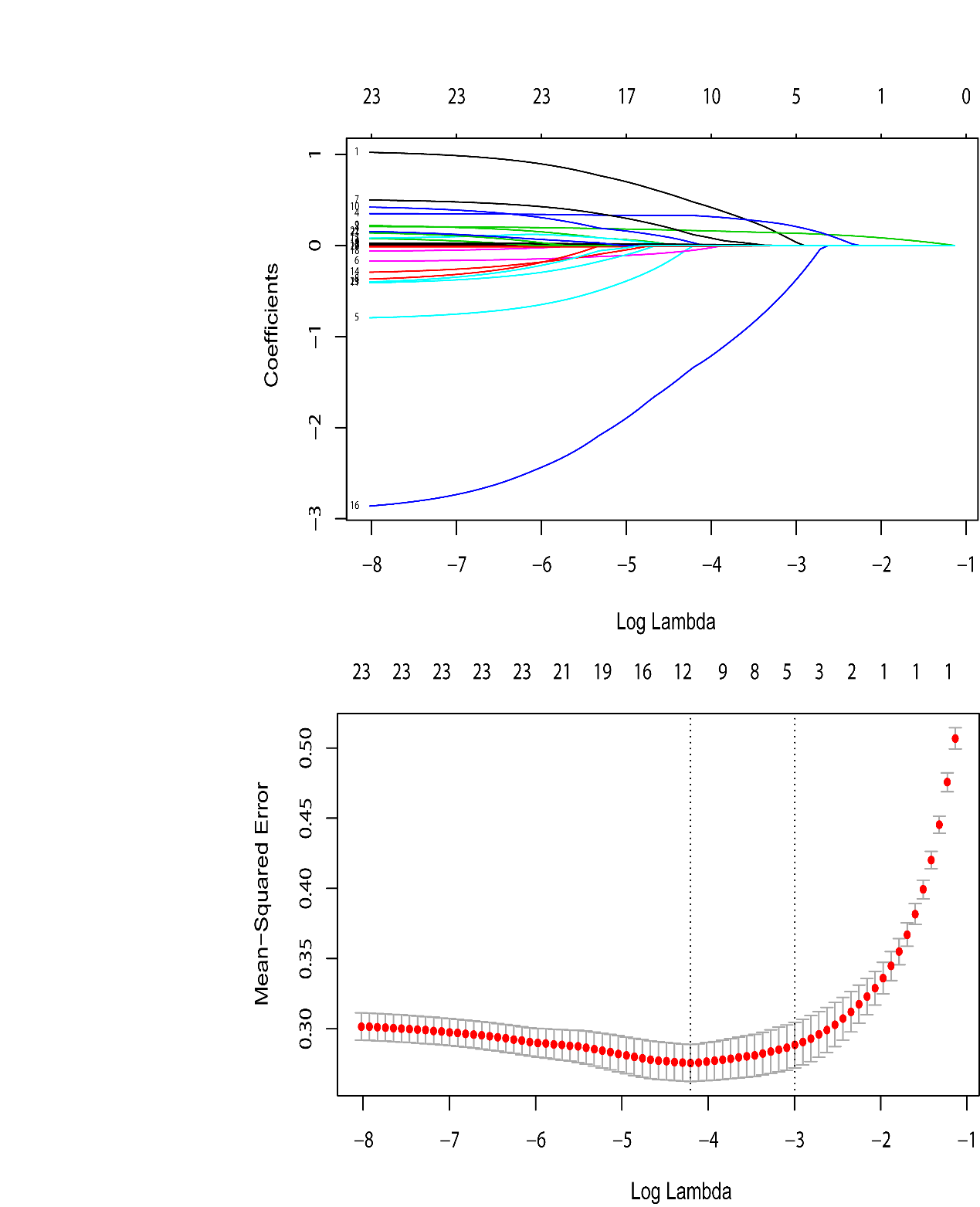


**Supplementary Figure 1.** Feature selection using LASSO regression. Identification of the optimal penalization coefficient (λ) in the LASSO model with 5-fold cross-validation and the 1 standard error (1-SE criteria). The dotted vertical line was drawn at the optimal λ at minimum criteria and 1 standard error (1-SE criteria). The model at 1-SE criteria was selected as the final model with 5 nonzero coefficients.

**Table S1. Correlation analysis between t-PA and other variables**

| **Variable** | **Correlation coefficient** | **P-value** |
| --- | --- | --- |
| Age | 0.310 | <0.001 |
| Gender | 0.084 | 0.149 |
| CAD | 0.110 | 0.060 |
| Hypertension | 0.042 | 0.476 |
| Smoking | 0.039 | 0.500 |
| FBG | 0.038 | 0.512 |
| Diabetes | -0.092 | 0.116 |
| eGFR | -0.218 | <0.001 |
| Calcium | -0.028 | 0.636 |
| Phosphorus | -0.078 | 0.182 |
| CRP | 0.198 | <0.001 |
| TC | 0.047 | 0.425 |
| TG | 0.111 | 0.058 |
| HDL-C | -0.139 | 0.017 |
| LDL-C | 0.040 | 0.494 |
| γ-GT | 0.171 | 0.003 |

**Supplementary Table 1.** FBG: fast blood glucose; HDL-C: high-density lipoprotein cholesterol; LDL-C: low-density lipoprotein cholesterol; TC: total cholesterol; TG: triglyceride; CRP: C-reactive protein, γ-GT: gamma-glutamyl transferase
